# Unraveling an extreme AT-rich and complex mitochondrial genome: the first complete mitogenome of the species-rich family Diaspididae (Hemiptera, Coccomorpha) and its evolutionary implications

**DOI:** 10.3897/zookeys.1272.178506

**Published:** 2026-03-02

**Authors:** Jun Deng, Lin Zhang, Wentao Ma, Hui Xiao, Congcong Lu, Xiaolei Huang

**Affiliations:** 1 State Key Laboratory of Agricultural and Forestry Biosecurity, College of Plant Protection, Fujian Agriculture and Forestry University, Fuzhou 350002, China College of Plant Protection, Fujian Agriculture and Forestry University Fuzhou China; 2 State Key Laboratory of Animal Biodiversity Conservation and Integrated Pest Management, Institute of Zoology, Chinese Academy of Sciences, Beijing 100101, China Institute of Zoology, Chinese Academy of Sciences Beijing China

**Keywords:** Armored scale insect, AT-rich mitogenome, Coccoidea, genome assembly pipeline, mitochondrial genome evolution

## Abstract

The assembly of insect mitochondrial genomes remains challenging in certain taxa due to extreme AT biases and long repetitive regions. The family Diaspididae (Hemiptera: Coccomorpha) exemplifies this difficulty; despite more than 2,700 described species worldwide, no complete mitogenome has been reported prior to this study. Here, the complete mitogenome is presented of *Aulacaspis yasumatsui*, assembled using a hybrid approach combining second- and third-generation sequencing platforms. The *A. yasumatsui* mitogenome is the longest identified to date among scale insects, spanning 21,273 bp. It exhibits several extraordinary features, most notably the highest AT content (92%) yet recorded in Insecta, extensive gene rearrangements, the absence of half of the tRNA genes, and the first identified ultra-long repeat region (> 5,000 bp) in Coccomorpha. The leg degeneration and loss observed in Diaspididae evolution may have contributed to the emergence of such an extreme mitogenome structure. Comparative analysis of sequencing strategies revealed substantial coverage bias in Illumina data, which was effectively mitigated by Oxford Nanopore long reads. We propose a standardized pipeline tailored for efficient and accurate assembly of highly AT-rich and structurally complex mitogenomes. This study provides both methodological insights and a valuable genomic resource for future evolutionary and comparative studies on Diaspididae and other taxa with challenging mitogenomes.

## Introduction

Mitochondrial genomes have been extensively utilized in various fields, including molecular evolution, phylogeny, genetic drift, and biogeography (Castro et al. 2002; Shao et al. 2003; Salvato et al. 2008; Ma et al. 2012). The research on the mitochondrial genome (mitogenome) of scale insects started relatively late; the first mitogenome of a scale insect, *Ceroplastes japonicus* Green, 1921, was obtained in 2019 (Deng et al. 2019). Currently, the mitogenome data of scale insects in the GenBank database remain very limited. It includes only 31 mitogenomes, representing 19 species of Coccomorpha across seven families (accessed December 2024). In insect mitogenomes, an AT bias exists, with an average AT content of approximately 76.5%. Mitogenomes of Hemiptera generally exhibit a pronounced AT bias in Insecta (Guo and Yuan 2016). Within the hemipteran suborder Sternorrhyncha, species of Coccomorpha exhibit exceptionally high AT content, with an average reaching 86.3%. Notably, the five insect mitogenomes with the highest AT content all belong to scale insects (Suppl. material 6). Second-generation sequencing (SGS) technology, such as Illumina, has been widely employed for the assembly of insect mitogenomes. However, due to its short read length and GC bias, it often results in uneven sequencing coverage, with AT-rich regions frequently underrepresented or entirely absent (Chen et al. 2013; Browne et al. 2020). This issue is particularly pronounced in certain insect taxa, such as scale insects, which possess extremely high AT content (≤ 85%). In these cases, other AT-rich regions within the mitogenome may further exacerbate assembly fragmentation. Consequently, the reconstruction of complete circular mitogenomes continues to face major assembly bottlenecks in these taxa.

Nevertheless, SGS technologies offered high coverage and accuracy, significantly accelerating the accumulation of mitogenome data (King et al. 2014; Peck et al. 2016). Currently, the assembly of mitogenomes using SGS data primarily relies on two approaches: De Bruijn graph (DBG)-based assembly and reference-guided assembly (Soorni et al. 2017). DBG-based tools, such as SPAdes (Bankevich et al. 2012), MEGAHIT (Li et al. 2015) and Velvet (Zerbino and Birney 2008), work by breaking sequencing reads into k-mers and constructing a graph based on the k-1 base overlap between adjacent k-mers, from which the optimal path is identified to reconstruct the genome sequence. This method is highly efficient for large-scale datasets but may encounter difficulties in handling repetitive sequences and in assembling circular mitogenome. Reference-guided tools, such as GetOrganelle (Jin et al. 2020) and NOVOPlasty (Dierckxsens et al. 2017), align sequencing reads to known reference genomes and iteratively extend and fill gaps to complete the assembly. This strategy is well-suited for highly conserved mitogenomes, but it may introduce biases when reference genomes are unavailable or when there is substantial sequence divergence, thereby requiring high-quality input data. Due to the extreme base composition bias and the lack of available reference genomes in scale insect mitogenomes, the assembly methods face significant challenges in achieving complete mitogenome reconstructions in certain lineages.

In recent years, third-generation sequencing (TGS) technologies, such as Oxford Nanopore Technologies (ONT) and PacBio SMRT sequencing, have been widely applied to the analysis of plant mitogenomes and chloroplast genomes (Li et al. 2025), owing to the larger size and higher structural complexity of these genomes. These technologies effectively address the challenges posed by such structural intricacies. In contrast to plants, animal mitogenomes are typically smaller and structurally simpler, and the application of TGS for mitogenome assembly in insects is still relatively limited. Assemblies based on long-read data typically employ the Overlap-Layout-Consensus (OLC) approach. Representative tools include Canu (Koren et al. 2017) and Flye (Kolmogorov et al. 2020), which construct contigs by identifying overlaps between reads and then generating the genome sequence based on the optimal layout. Unlike SGS, TGS generates long reads ranging from tens to hundreds of kilobases, which greatly facilitate the assembly of repetitive regions and those with high AT content (Hu et al. 2021). A major limitation of TGS is its relatively high cost compared to SGS. For example, ONT sequencing costs approximately 600 USD per sample, whereas SGS typically costs around 100 USD for a data yield of 20 GB (based on current quotations from sequencing service providers). Therefore, for large-scale studies aimed solely at obtaining mitogenomes, TGS may not be a cost-effective option.

In most insects, the mitogenome typically retains the ancestral gene order. However, in certain insects, gene rearrangements have occurred in the mitogenome, mainly through mechanisms such as inversion, translocation, and gene shuffling (Shao et al. 2001; Cameron 2024). Studies have shown that an increased nucleotide substitution rate in insect mitogenomes may promote a higher frequency of gene rearrangements (Shao et al. 2003; Wang et al. 2021; Liu et al. 2023). Most hemipteran families with available mitogenome retain the ancestral insect gene order (Wang et al. 2015). However, previous studies have found that the mitochondrial evolutionary rate in scale insects (Coccomorpha) is significantly higher than in other Hemiptera (Lu et al. 2023). In Aclerdidae and Coccidae the *nad2* gene is translocated, while the *trnY* gene undergoes both translocation and inversion. Even more remarkably, in Tachardiidae (= Kerriidae) the gene cluster *nad1-trnL1-rrnL-trnV-rrnS* has translocated from downstream of *cytb* to a position after *nad4L* (Xu et al. 2023). Additionally, scale insects exhibit a rare phenomenon of extensively truncated tRNAs, where the absence of the dihydorouridine (DHU) or TΨC (T) arm in tRNA genes leads to an atypical cloverleaf secondary structure, complicating tRNA annotation (Lu et al. 2020). Furthermore, tRNA gene loss has been documented in coccoid mitogenomes: the *trnV* gene is undetectable in the Eriococcidae*Antecerococcus theydoni* Hall, 1935 and *Acanthococcus coriaceus* Miller & Gimpel, 1996, while the *trnC* gene is likewise absent in *Albotachardina sinensis* Zhang, 1922 (Kerriidae) (Xu et al. 2023).

**Figure 1.**
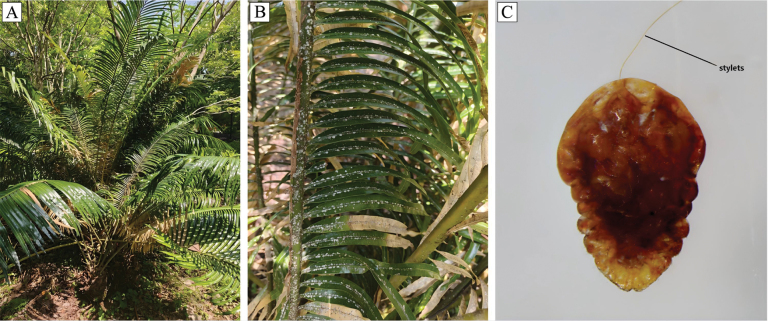
Damage symptoms and morphological characteristics of *Aulacaspis yasumatsui*. **A, B**. Infestation symptoms of *A. yasumatsui* on *Cycas szechuanensis*; **C**. Ventral morphological characteristics of *A. yasumatsui*.

Diaspididae belong to the order Hemiptera and the infraorder Coccomorpha. To date, Diaspididae comprises 419 genera and 2,714 species, accounting for 31.7% of the total species within Coccomorpha, making it the most species-rich family in the superfamily (García Morales et al. 2016). These insects feed on a wide range of host plants, with many species recognized as significant agricultural and forestry pests (Fig. 1). The nymphs are mobile during their first stages, using their legs to move and feed on host plants. At the second instar, the legs begin to degenerate, sexual dimorphism begins to develop, and the adult stage exhibits distinct sexual differences (Boratyński 1953; Podsiadło 2019). Male adults are winged, active, do not feed, and have a short lifespan, whereas female adults possess a hardened protective scale, have degenerated legs, remain sessile on host plants, and have a relatively longer lifespan (Normark et al. 2019). In addition, since Diaspididae are easily parasitized, the samples may be contaminated with parasitoid wasp genomes during sequencing and assembly, leading to potential issues with genomic contamination. In 2020, the complete mitogenome of *Unaspis yanonensis* (Liu et al. 2020) was published but later confirmed to belong to a parasitic wasp rather than a diaspidid (Xu and Wu 2022). The absence of mitogenome data for such a large and economically important group of pests in public databases may indicate the complexity of genome assembly and the unique structural characteristics of their mitogenomes.

Efficient and cost-effective acquisition of mitogenomes from a large number of taxa with challenging assemblies remains a pressing issue, particularly for advancing studies on mitogenome diversity, evolution, and phylogenetics. Using scale insects of the family Diaspididae as a representative case, this study addresses this gap through three core objectives: to establish a standardized pipeline that integrates both SGS and TGS technologies, revealing the structural complexity of the first complete mitogenome of Diaspididae, and providing an evolutionary perspective on its possible origins.

## Materials and methods

### Sampling, DNA extraction, and sequencing

Specimens of *Aulacaspis yasumatsui* Takagi, 1977 were collected from *Cycas szechuanensis* at Fujian Agriculture and Forestry University in Fuzhou, Fujian, China (26°04'27"N, 119°17'47"E) and preserved in 95% ethanol and stored at -80 °C. The species was then identified based on morphological characteristics and molecular analysis. Before DNA extraction, the wax covering of *A. yasumatsui* was removed. A total of 1 g of the processed samples was collected, and genomic DNA was extracted from the entire bodies of the specimens using the DNeasy Blood & Tissue Kit (Qiagen, Hilden, Germany) following the manufacturer’s protocol. The extracted DNA was sent to Novogene Company (Tianjin, China) for SGS and TGS. For SGS, DNA was used to construct sequencing libraries according to the manufacturer’s protocol. The library was sequenced on the Illumina NovaSeq 6000 platform with paired-end reads of 150 bp, generating 21 Gb of clean data after quality assessment. The extracted DNA was split into two samples for SGS and TGS, respectively. For TGS, the specimen was sequenced using ONT on the PromethION platform with R9.4.1 flow cells and LSK110 kits, achieving a sequencing depth of 30× and generating approximately 50 Gb of clean data.

### Mitogenome assembly and quality control

For the SGS data, we employed two assembly methods: de novo assembly and seed-extend-based de novo assembly. First, de novo assembly of the reads was performed using Megahit v1.2.9 (Li et al. 2015) and SPAdes v3.15.5 (Bankevich et al. 2012). Scale insect mitogenomes from GenBank were used as query sequences, and BLAST v2.5.0 (Altschul et al. 1990) was employed to search the de novo assembled sequence database to identify mitogenome contigs. Second, NOVOPlasty v4.3.3 (Dierckxsens et al. 2017) was used to assemble the mitogenome sequence, with *cox1* sequence (confirmed by BLAST v2.5.0 with an E-value of 0) as the seed and the K-mer set to 27. Geneious v2023.0.1 (Kearse et al. 2012) was used to assemble the extracted contigs into a single sequence. For the ONT long reads, Minimap2 v2.26 (Li 2018) was used to align the ONT reads of *A. yasumatsui* to the assembled mitogenome fragments from Illumina data and the mitogenomes of closely related species. Samtools v1.18 (Li et al. 2009) was used to extract the aligned reads and convert them into FASTQ format. Canu v2.2 (Koren et al. 2017) was then used to assemble the aligned reads and detect circularization. Medaka v1.11.1 was employed for base correction of the mitogenome of *A. yasumatsui* (https://github.com/nanoporetech/medaka, medaka_consensus -m r1041_e82_400bps_hac_g615). Unless otherwise specified, all software was run with default parameters. The obtained genomic data were subjected to quality control using the LongQC software (Fukasawa et al. 2020). The detailed workflow is shown in Suppl. material 7.

### Mitochondrial genome annotation

Primary annotation was conducted using the MITOS Web Server (MITOS2) (Bernt et al. 2013) with the settings “Reference: RefSeq 63 metazoa”, “Genetic Code: 5 Invertebrate”, and advanced option “AI lab”. The sequences of 13 protein-coding genes (PCGs) and two ribosomal RNA genes (rRNAs) from Coccomorpha were downloaded from NCBI to verify and redefine gene boundaries through homology alignment. For tRNAs that were not annotated during the initial annotation, tRNA annotation was performed following the method described by Lu et al. (2020), which involves identifying tRNAs in the non-coding region and combining the results of ARWEN (Laslett and Canbäck 2008) and MITOS2. The secondary structure of tRNA genes was drawn using ViennaRNA v2.7.0 (Lorenz et al. 2011). *Aulacaspis yasumatsui* mitogenome structure was visualized using the CGview (Grant and Stothard 2008) online server. Additionally, Tandem Repeats Finder (TRF) (Benson 1999) online tool was used to search for tandem repeat sequences in the control region and repeat region of the *A. yasumatsui* mitogenome.

### Mitochondrial read coverage visualization and circularization validation

Both Illumina and ONT clean data were mapped to the *A. yasumatsui* mitogenome using Minimap2 v2.26 (Li 2018). The alignment results were processed using Samtools v1.18 (Li et al. 2009) for format conversion and sorting, followed by the extraction of coverage data. Finally, the coverage map was generated using CGview (Grant and Stothard 2008) online server to visualize the sequencing depth distribution across the entire genome. The BAM file was viewed through IGV v2.17.1 (Thorvaldsdóttir et al. 2013) to confirm whether reads span the entire region in the control and repeat region.

The size of the mitogenome was determined by electrophoresis to verify its consistency with the genome sequencing results, thereby confirming whether it is circular. The procedure was as follows: After removing the wax covering, 200 mg of the sample was collected, and mitochondrial DNA was extracted using the Solarbio Mitochondrial Extraction Kit. A 1% agarose gel electrophoresis was performed for detection, and the Genstar D20K marker was used for labeling. All experimental steps were conducted strictly according to the manufacturer’s instructions.

### Comparative mitogenomic analyses

A total of 17 mitogenomes from 8 families of Coccomorpha were included for the subsequent comparative analysis, combining mitogenomes downloaded from NCBI and the mitogenome of *A. yasumatsui* (Suppl. material 1). Gene orders were visualized using the online software Interactive Tree Of Life (iTOL) v5 (Letunic and Bork 2021), with different types of genes depicted in distinct colors. The AT content, GC skew = [G-C] / [G + C], and AT skew = [A-T] / [A + T] were calculated using PhyloSuite v2.2.1 (Zhang et al. 2020). The relative synonymous codon usage (RSCU) of the PCGs of mitogenome was extracted using PhyloSuite v2.2.1. The RSCU results were plotted using ggplot2.

### Phylogenetic analysis

The phylogenetic relationships among 17 mitogenomes of Coccomorpha were constructed with three outgroups (*Adelges tsugae*, MT263947.1; *Nippolachnus piri*, OL069343.1; *Aphis aurantii*, OM350401.1). Extracted 13 PCGs and 2 rRNAs (PCG123rRNA) as a dataset, the all sequences were aligned using MAFFT v7 with the “auto” strategy (Katoh and Standley 2013) in codon alignment mode. Gap sites were removed using trimAI with the “strictplus”. All sequences were concatenated in PhyloSuite v2.2.1. ModelFinder (Kalyaanamoorthy et al. 2017) was utilized to select the best-fit model using the Bayesian Information Criterion (BIC). Two approaches were employed: maximum likelihood (ML) analysis and the PhyloBayes CAT-GTR model, conducted using IQ-TREE v 2.3.6 (Nguyen et al. 2015) and PhyloBayes (Lartillot et al. 2013), respectively. PhyloBayes CAT-GTR model is assumed to be particularly well suited to resolve artefacts caused by long-branch attraction and site-specific compositional heterogeneity (Lartillot et al. 2007; Whelan and Halanych 2017). The ML analyses were conducted with 1,000 replicates and the PhyloBayes analyses with parameters set to ngen = 1,000,000, samplefreq = 100, and burninfrac = 0.25. The resulting phylogenetic trees were visualized using FigTree.

## Results

### Genome organization and base composition

**Figure 2.**
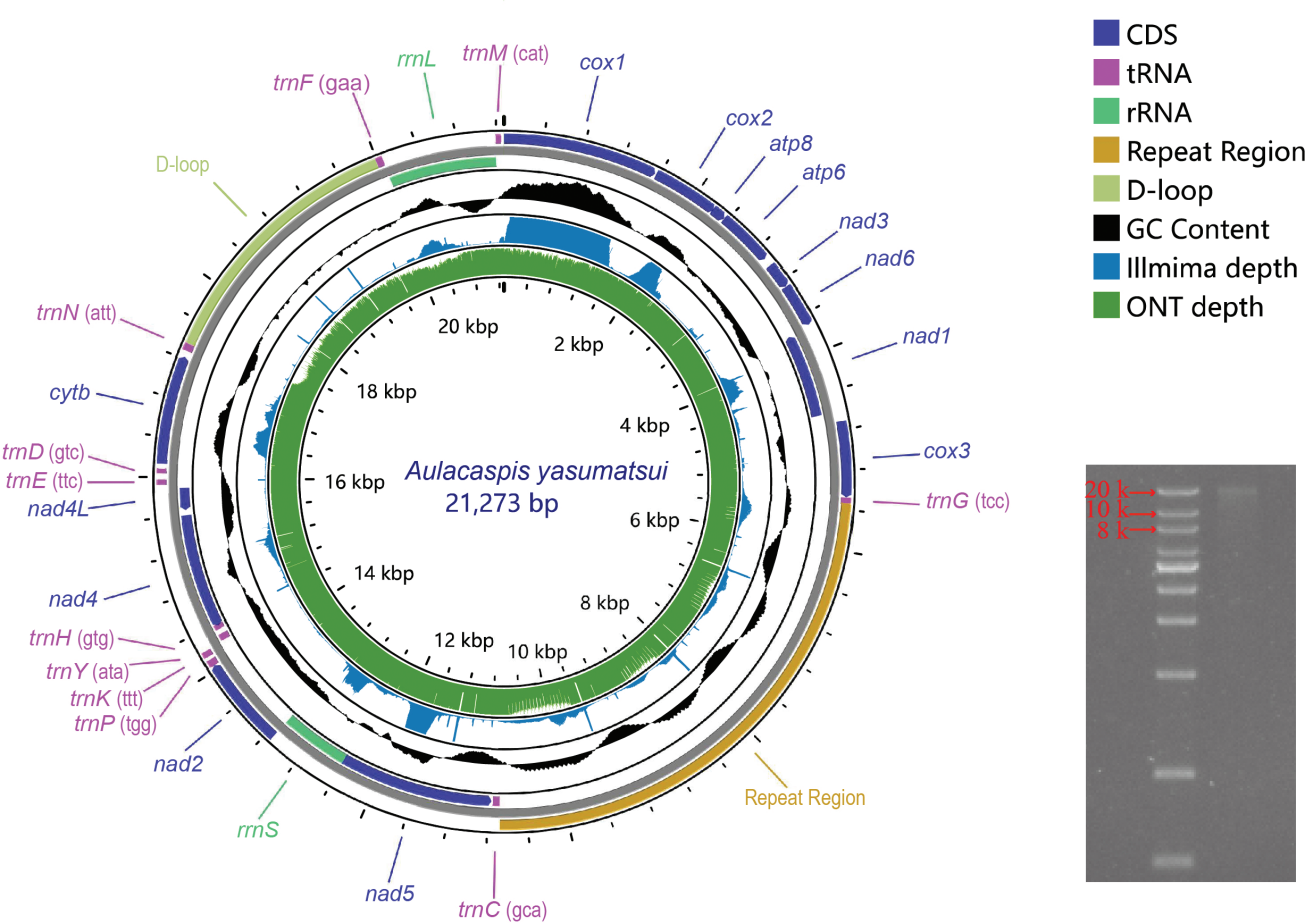
Structure of the *A. yasumatsui* mitochondrial genome and gel electrophoresis image. The maximum sequencing depth displayed for Illumina was capped at 500, while the maximum sequencing depth displayed for ONT was 6,000.

The SGS assembly results yielded only two mitogenome contigs, covering a portion of the *cox1* gene to part of the repeat region (located between 323 bp and 5,646 bp of the genome) and a portion of the *rrnS* gene to part of the control region (located between 13,002 bp and 17,538 bp of the genome). Based on TGS, we successfully obtained the complete mitogenome of *A. yasumatsui*, which was 21,273 bp in length, significantly larger than the mitogenomes of other scale insects (Fig. 2). The mitogenome sequence has been deposited in GenBank under accession number PP034407. Base composition analysis revealed AT bias of 0.118, GC bias of -0.144, and AT content of 92.1%. The genome contained 26 mitochondrial genes (13 PCGs, 11 tRNA genes, 2 rRNA genes), a control region, and a repeat region (Fig. 2, Suppl. material 2). In the mitogenome of *A. yasumatsui*, the genes *nad1*, *nad4*, *nad4L*, *nad5*, *rrnS*, *rrnL*, *trnC*, *trnY*, and *trnH* are located on the N strand, while the other genes are on the J strand. The lengths of the PCGs, rRNA genes, and control region were 10,377 bp, 1,861 bp, and 2,635 bp, respectively. Most PCGs initiate with the standard start codon (ATN), except for *cox1* and *nad3*, which start with TTA and AAA, respectively, and all PCGs terminate with the standard stop codon (TAA).

### Transfer RNA

The mitogenome of *A. yasumatsui* is missing half of the tRNA genes, with only 11 tRNA genes annotated, ranging in length from 51 bp (*trnD*) to 73 bp (*trnC*) (Suppl. material 2). All tRNA genes were predicted and their secondary structures were drawn (Suppl. material 8), with only *trnF* identified through sequence alignment with corresponding genes from other scale insects. The remaining 10 genes were predicted using MITOS2 and ARWEN. Among them, *trnC*, *trnF*, *trnK*, *trnN*, *trnP*, *trnY* gene exhibit the typical cloverleaf secondary structure.

### Control region and repeat region

The mitochondrial control region of *A. yasumatsui* contains 2,635 bp, accounting for 12.4% of the total genome length, with an AT content of 93.2%. It is located between the genes *trnN* and *trnF*. The predicted tandem repeat sequences in the control region are presented in Suppl. material 3. A total of 11 tandem repeat sequences were identified, with the longest segment located in the 17,421–17,746 bp region of the genome, where a 19 bp fragment is repeated 17.3 times. The mitogenome of *A. yasumatsui* also contains a repeat region located between *trnC* and *trnG*, with an AT content of 92.1% and length of 5,149 bp, accounting for 24.2% of the total genome length. The predicted tandem repeat sequences in this region are shown in Suppl. material 4. A total of 49 tandem repeat sequences were found, with the longest segment located in the 6,695–10,618 bp region, where a 54 bp fragment is repeated 74 times.

### Mitogenome read coverage

**Figure 3.**
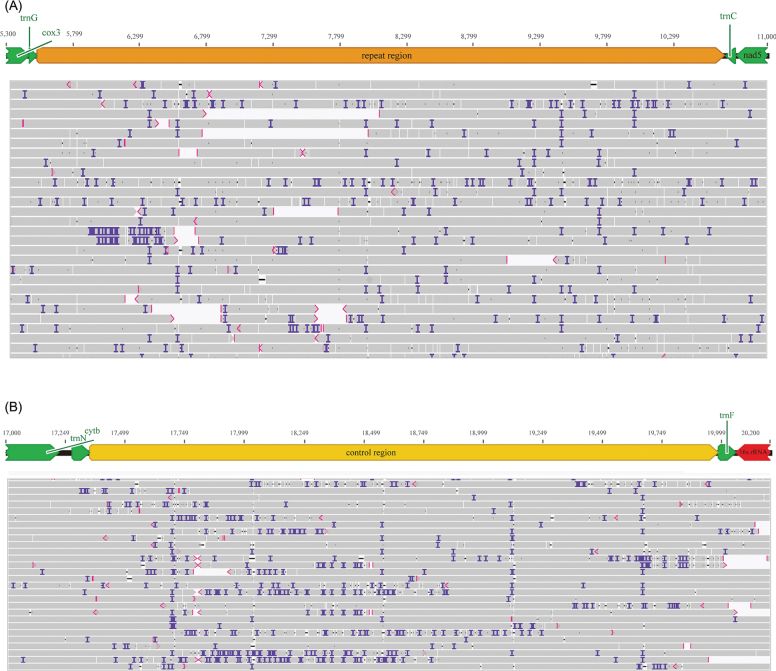
ONT reads coverage map of the repeat and control regions. Purple areas represent inserted bases.

The SGS depth distribution shows a clear GC bias, with regions of higher GC content exhibiting significantly higher read coverage, while regions with lower GC content (such as the control region) show a marked decrease in coverage, even exhibiting noticeable coverage gaps (Fig. 2). In contrast, although ONT long-read sequencing also achieved a high coverage depth of up to 30,000 reads in the *cox1* gene region, it exhibited a more uniform coverage across the remainder of the genome (Fig. 2). Moreover, several reads successfully spanned challenging regions such as the control region and repeat region (Fig. 3). The electrophoresis results confirmed that the mitogenome size is consistent with the sequencing data, verifying its circular nature (Fig. 2).

### Codon usage

**Figure 4.**
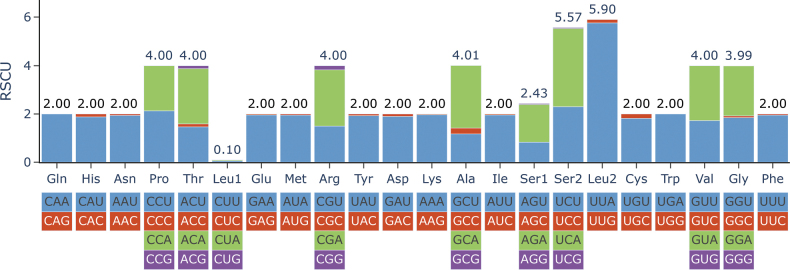
Relative synonymous codon usage (RSCU) in the mitogenomes of *A. yasumatsui*. Codon families were labeled on the x-axis, and values on top of the bars denoted amino acid usage. Stop codons were not included.

The 13 PCGs of *A. yasumatsui* mitogenome contain a total of 49 codons. The five most frequently used codons are AUU (Ile) 524 times; AUA (Met) 482 times; UUU (Phe) 472 times; AAU (Asn) 392 times; and AAA (Lys) 196 times (Suppl. material 5). All of these codons are composed of A and U. *A. yasumatsui* exhibits stronger codon preferences, tending to use codons with higher A/T content. Additionally, from the comparative analysis of RSCU, it can be observed that all amino acids prefer codons ending with A/T bases, with a higher proportion in the stacked plot (Fig. 4).

### Gene rearrangement

**Figure 5.**
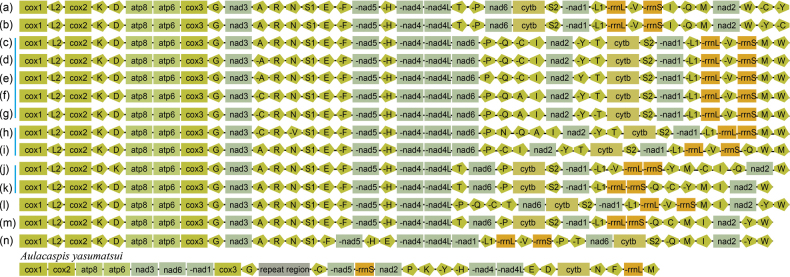
Gene arrangement of mitochondrial genome in Coccomorpha. (**a**), (**b**), (**c, d, e, f, g**), (**h, i**), (**j, k**), (**l**), (**m**), (**n**) belong to the different family, respectively. Genes with negative signs (-) were located on the minor Strand, and other genes are located on the major Strand. Different gene types were displayed using distinct shapes and colors. Labels (**a–n**) correspond to: **a**. Ancestral insect; **b**. *Matsucoccus matsumurae* (Matsucoccidae); **c**. *Didesmococcus koreanus* (Coccidae); **d**. *Parasaissetia nigra* (Coccidae); **e**. *Coccus hesperidum* (Coccidae); **f**. *Saissetia coffeae*, *Ceroplastes floridensis*, *Ceroplastes japonicus*, and *Ceroplastes rubens* (Coccidae); **g**. *Ericerus pela* (Coccidae); **h**. *Aclerda takahashii* (Aclerdidae); **i**. *Nipponaclerda biwakoensis* (Aclerdidae); **j**. *Apiomorpha munita* (Eriococcidae); **k**. *Acanthococcus coriaceus* (Eriococcidae); **l**. *Phenacoccus manihoti* (Pseudococcidae); **m**. *Antecerococcus theydoni* (Cerococcidae); **n**. *Albotachardina sinensis* (Kerriidae).

The mitogenome of scale insects exhibited highly variable gene order (Fig. 5). Compared to other scale insects, *A. yasumatsui* also exhibits significant rearrangements. In *A. yasumatsui*, there are conserved gene cluster such as *atp8-atp6*, *cox3-trnG*, and *trnH-nad4-nad4l*, while other genes showed significant rearrangement and loss of tRNA genes. By comparing the gene orders with ancestral insect mitochondria, we found that *nad3*, *nad6*, and *nad1* have been rearranged and inserted between *atp6* and *cox3*, forming the *atp6-nad3-nad6-nad1-cox3* PCG cluster. The position of *nad2* had also shifted, forming the *nad5-nad2-nad4-nad4L* PCG cluster. The positions of *rrnS* and *rrnL* also underwent significant adjustments. Additionally, the *A. yasumatsui* mitogenome has lost about half of its tRNA genes, and the remaining tRNAs exhibit notable changes in their order, especially *trnP*, *trnK*, *trnY*, *trnC*, *trnE*, *trnD*, *trnN*, and *trnF*, which have undergone rearrangements.

### Phylogenetic analysis

**Figure 6.**
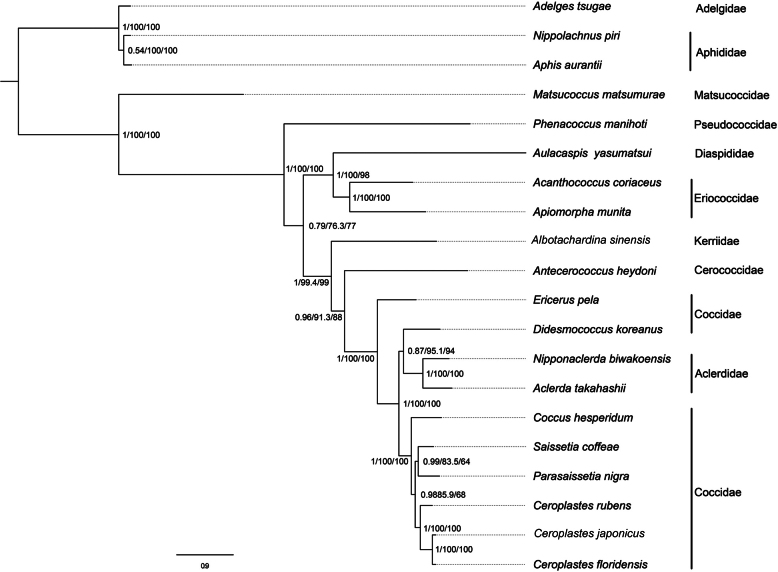
Phylogenetic tree of Coccomorpha inferred from the PhyloBayes and ML analyses based on the PCG123rRNAs dataset. Node labels indicate, in order, bootstrap support values, SH-aLRT support values, and ultrafast bootstrap support values.

We conducted phylogenetic analyses based on PCG123rRNA from 17 species representing eight families of scale insects. Both ML and PhyloBayes analyses yielded consistent topologies with high node support values (Fig. 6). Within Coccomorpha, Matsucoccidae as the basal lineage, with the remaining families forming a single clade, referred to as the neococcoid clade. Within the neococcoid clade, Pseudococcidae was positioned as the basal lineage. Eriococcidae and Diaspididae formed a sister group relationship, while the relationships among the remaining families were resolved as (Kerriidae + (Cerococcidae + (Aclerdidae + Coccidae))). Due to the nesting of Aclerdidae within Coccidae, Coccidae was found to be paraphyletic. Additionally, Aclerdidae and *Didesmococcus koreanus* were recovered as sister taxa.

## Discussion

The assembly of mitogenomes in scale insects has long posed a significant challenge, impeding evolutionary and phylogenetic studies reliant on these data. Our successful acquisition of the complete mitogenome of *A. yasumatsui*, the first for the family Diaspididae, underscores the efficacy of a hybrid sequencing strategy in overcoming the obstacles presented by extreme genomic features. The *A. yasumatsui* mitogenome is remarkable, holding the record for the highest AT content (92.1%) among known insects. Notably, scale insects occupy the top five positions for highest AT content in GenBank, suggesting a shared evolutionary trajectory toward nucleotide composition extremity within Coccomorpha. While previous studies have obtained complete mitogenomes for some scale insects using NGS (Deng et al. 2019; Lu et al. 2020; Lu et al. 2023; Xu et al. 2023), our study highlights a critical limitation of SGS: pronounced coverage gaps in hyper-AT-rich regions, leading to fragmented assemblies (Fig. 2). In contrast, ONT long-read sequencing provided uniform coverage and successfully spanned complex repetitive regions, proving indispensable for assembling the most challenging mitogenomes, such as the exceptionally long and AT-rich genome reported here.

The ultra-long repeat region (>5 kb) identified in *A. yasumatsui* is a novel finding within Coccomorpha. Such extensive repetitive regions, while uncommon, have been documented in other hemipterans, such as aphids and thrips, where they are a primary driver of mitogenome size expansion (Atray et al. 2015; Liu et al. 2017; Zhang et al. 2021). The evolutionary origin of these repeats in scale insects remains enigmatic. They may represent ancestral traits that were secondarily lost in other lineages, or they could have arisen independently through mechanisms like slipped-strand mispairing, a common cause of mitochondrial repeat proliferation (Aird et al. 2011). The limited availability of complete scale insect mitogenomes currently precludes a definitive conclusion, highlighting the need for broader sampling.

Beyond its size and base composition, the *A. yasumatsui* mitogenome exhibits an extraordinary degree of structural plasticity. While most Hemiptera retain a highly conserved gene order at the family level, Coccomorpha is a notable exception. In *A. yasumatsui*, conserved gene clusters are almost entirely absent relative to other Coccomorpha species, and both rRNA genes have undergone substantial translocations (Fig. 5). This extensive rearrangement aligns with the significantly elevated mitochondrial evolutionary rates reported for scale insects (Lu et al. 2023). A general correlation between high nucleotide substitution rates and increased frequency of gene rearrangements has been established across insects (Shao et al. 2001; Cameron 2024). The accelerated evolutionary rate in scale insects may be linked to their unique life-history traits. A recent bilaterian-wide analysis found that endoparasitic lifestyle is correlated with the highest mitochondrial evolutionary rates, while sedentary ectoparasites also exhibit elevated rates compared to free-living relatives (Jakovlić et al. 2023). Notably, the scale insect *Didesmococcus koreanus* (Coccidae) was identified as having one of the highest rates among all insects (Jakovlić et al. 2023). Adult female diaspidids, like many scale insects, are sessile, with degenerated or lost legs, and exhibit high levels of adaptation to a parasitic lifestyle on plants (Williams 1992; Ben-Dov 2006). This reduction in locomotory capacity and effective population size may relax purifying selection, permitting the accumulation of both point mutations and large-scale structural changes.

The complexity of scale insect mitogenomes is further exemplified by the pervasive reduction and loss of tRNA genes. In *A. yasumatsui*, approximately half of the typical tRNA complement is absent. While annotation challenges can lead to underestimation, our integrated approach of software prediction and manual curation provides strong evidence for the genuine loss of these genes. This phenomenon is not without precedent; mitochondrial tRNA gene loss and functional replacement by nuclear-encoded counterparts is a well-documented evolutionary process across diverse eukaryotes (Adams and Palmer 2003; Zhou et al. 2022). The extensive tRNA gene loss and genome rearrangement observed in *A. yasumatsui* may therefore represent an advanced stage of mitogenome streamlining, potentially driven by their extreme host dependence and sessile lifestyle. The mitochondrial genes whose functions are supplanted by the nuclear genome, leading to a more minimal and deranged mitochondrial genetic system.

In light of these challenges, we established an efficient and cost-effective assembly workflow. This pipeline strategically leverages the affordability of SGS while reserving TGS for the most recalcitrant samples. Its key advantages include: 1) Multi-tool collaborative assembly: Utilizing algorithms with different strengths (e.g., MEGAHIT and NOVOPlasty) for efficient assembly and mutual validation. 2) Optimized TGS read utilization: Using confirmed partial mitogenome sequences as references to selectively enrich for mitochondrial long reads, drastically accelerating the assembly process. 3) Strict contamination control: A critical step given the known issue of parasitoid contamination in scale insect samples, ensuring the authenticity of the assembled genome.

## Conclusions

In summary, this study deciphers the first complete mitogenome of the diaspidid insect *A. yasumatsui*, revealing an extraordinary architecture characterized by an extreme AT bias (92%), widespread tRNA gene loss, extensive gene rearrangements, and a 5,149-bp ultra-long repeat region. These findings suggest that the mitogenome architecture of Diaspididae may reflect unique evolutionary trajectories shaped by their sedentary lifestyle and ecological specialization. By integrating third-generation and second-generation sequencing technologies, we effectively overcame the challenges associated with highly AT-rich content and ultra-long repetitive regions in mitogenomes. Our work thus provides not only a robust methodological framework for assembling complex insect mitogenomes, but also contributes valuable genomic resources for future investigations into the mechanisms driving extreme structural variation and mitochondrial evolution in Coccomorpha and other derived insect lineages.

## Additional information

### Use of AI

No use of AI was reported.

### Author ORCIDs

Jun Deng https://orcid.org/0000-0002-5390-6362

Lin Zhang https://orcid.org/0009-0007-5393-2177

Wentao Ma https://orcid.org/0009-0007-1285-1602

Hui Xiao https://orcid.org/0000-0002-8979-3793

Congcong Lu https://orcid.org/0000-0002-2219-7196

Xiaolei Huang https://orcid.org/0000-0002-6839-9922

### Data availability

The mitogenome data of *A. yasumatsui* is available in the GenBank database under the accession number PP034407.

## Supplementary materials

Supplementary material 1 Mitochondrial genomic information of Coccomorpha Data type xlsx https://binary.pensoft.net/file/1552194This dataset is made available under the Open Database License (http://opendatacommons.org/licenses/odbl/1.0/). The Open Database License (ODbL) is a license agreement intended to allow users to freely share, modify, and use this Dataset while maintaining this same freedom for others, provided that the original source and author(s) are credited. Jun Deng, Lin Zhang, Wentao Ma, Hui Xiao, Congcong Lu, Xiaolei Huang

Supplementary material 2 Annotation of *Aulacaspis yasumatsui* mitogenome Data type xlsx https://binary.pensoft.net/file/1552195This dataset is made available under the Open Database License (http://opendatacommons.org/licenses/odbl/1.0/). The Open Database License (ODbL) is a license agreement intended to allow users to freely share, modify, and use this Dataset while maintaining this same freedom for others, provided that the original source and author(s) are credited. Jun Deng, Lin Zhang, Wentao Ma, Hui Xiao, Congcong Lu, Xiaolei Huang

Supplementary material 3 Tandem repeat sequences in the mitochondrial control region of *Aulacaspis yasumatsui* Data type xlsx https://binary.pensoft.net/file/1552196This dataset is made available under the Open Database License (http://opendatacommons.org/licenses/odbl/1.0/). The Open Database License (ODbL) is a license agreement intended to allow users to freely share, modify, and use this Dataset while maintaining this same freedom for others, provided that the original source and author(s) are credited. Jun Deng, Lin Zhang, Wentao Ma, Hui Xiao, Congcong Lu, Xiaolei Huang

Supplementary material 4 Tandem repeat sequences in the mitochondrial repeat region of *Aulacaspis yasumatsui* Data type xlsx https://binary.pensoft.net/file/1552197This dataset is made available under the Open Database License (http://opendatacommons.org/licenses/odbl/1.0/). The Open Database License (ODbL) is a license agreement intended to allow users to freely share, modify, and use this Dataset while maintaining this same freedom for others, provided that the original source and author(s) are credited. Jun Deng, Lin Zhang, Wentao Ma, Hui Xiao, Congcong Lu, Xiaolei Huang

Supplementary material 5 Codon usage of protein-coding genes in *Aulacaspis yasumatsui* mitogenome Data type xlsx https://binary.pensoft.net/file/1552198This dataset is made available under the Open Database License (http://opendatacommons.org/licenses/odbl/1.0/). The Open Database License (ODbL) is a license agreement intended to allow users to freely share, modify, and use this Dataset while maintaining this same freedom for others, provided that the original source and author(s) are credited. Jun Deng, Lin Zhang, Wentao Ma, Hui Xiao, Congcong Lu, Xiaolei Huang

Supplementary material 6 Scatter plot of mitochondrial genome length and AT content in insects Data type jpg Explanation note Data obtained from the NCBI RefSeq database in September 2024; red dots represent scale insect data. https://binary.pensoft.net/file/1552199This dataset is made available under the Open Database License (http://opendatacommons.org/licenses/odbl/1.0/). The Open Database License (ODbL) is a license agreement intended to allow users to freely share, modify, and use this Dataset while maintaining this same freedom for others, provided that the original source and author(s) are credited. Jun Deng, Lin Zhang, Wentao Ma, Hui Xiao, Congcong Lu, Xiaolei Huang

Supplementary material 7 Workflow chart Data type png Explanation note Workflow chart for mitochondrial genome assembly using high-throughput sequencing technology. https://binary.pensoft.net/file/1552200This dataset is made available under the Open Database License (http://opendatacommons.org/licenses/odbl/1.0/). The Open Database License (ODbL) is a license agreement intended to allow users to freely share, modify, and use this Dataset while maintaining this same freedom for others, provided that the original source and author(s) are credited. Jun Deng, Lin Zhang, Wentao Ma, Hui Xiao, Congcong Lu, Xiaolei Huang

Supplementary material 8 Secondary structure of tRNA genes in *A. yasumatsui* mitochondrial genome Data type jpg https://binary.pensoft.net/file/1552201This dataset is made available under the Open Database License (http://opendatacommons.org/licenses/odbl/1.0/). The Open Database License (ODbL) is a license agreement intended to allow users to freely share, modify, and use this Dataset while maintaining this same freedom for others, provided that the original source and author(s) are credited. Jun Deng, Lin Zhang, Wentao Ma, Hui Xiao, Congcong Lu, Xiaolei Huang
