# Supplementary figures and images for: Unraveling an extreme AT-rich and complex mitochondrial genome: the first complete mitogenome of the species-rich family Diaspididae (Hemiptera, Coccomorpha) and its evolutionary implications
Source: Zookeys. 2026 Mar 2;1272:47–66. doi: 10.3897/zookeys.1272.178506 (PMC12973041; doi:10.3897/zookeys.1272.178506)

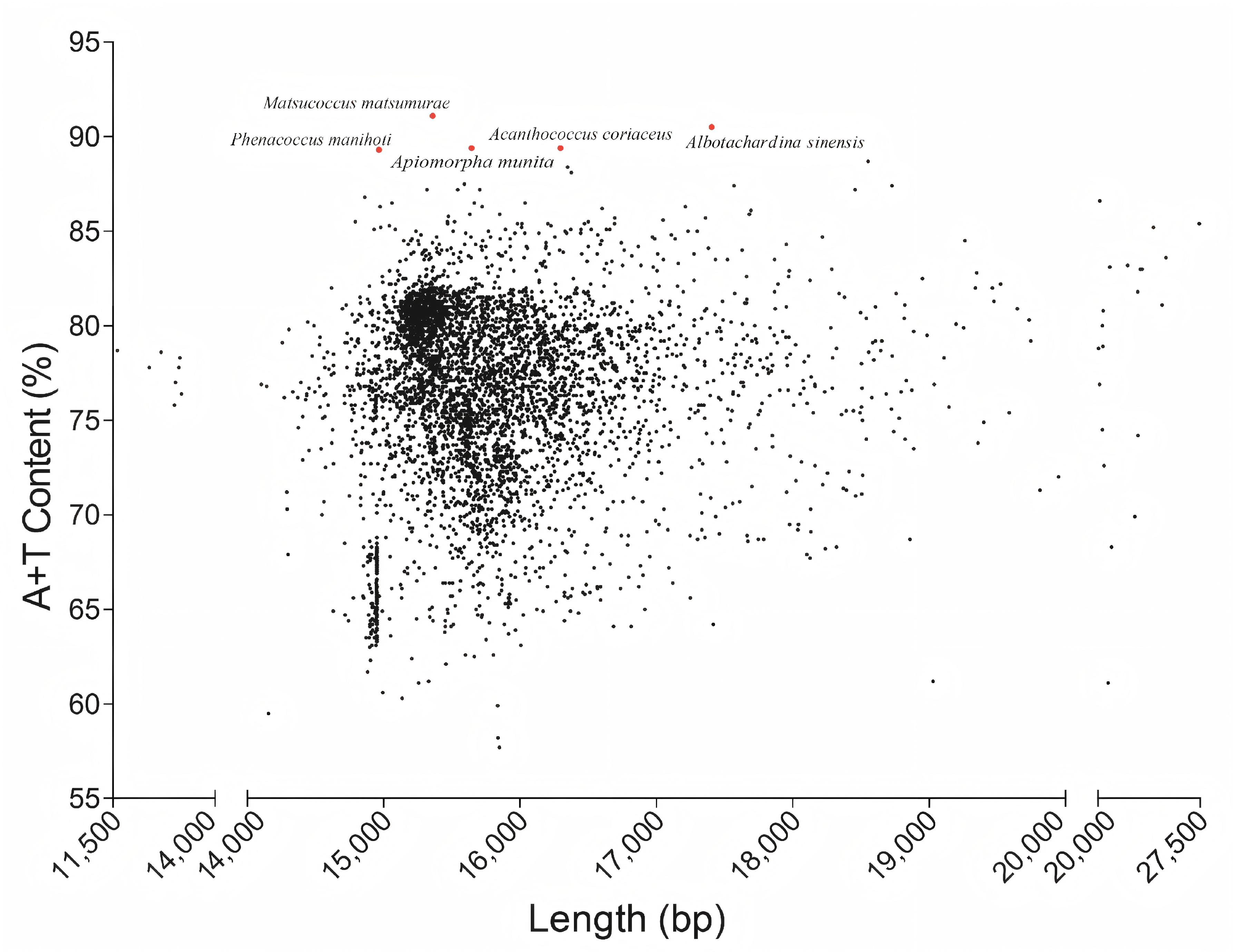

Supplement: Supplementary material 6 — Scatter plot of mitochondrial genome length and AT content in insects [file zookeys-1272-047_article-178506__-s006.jpg]

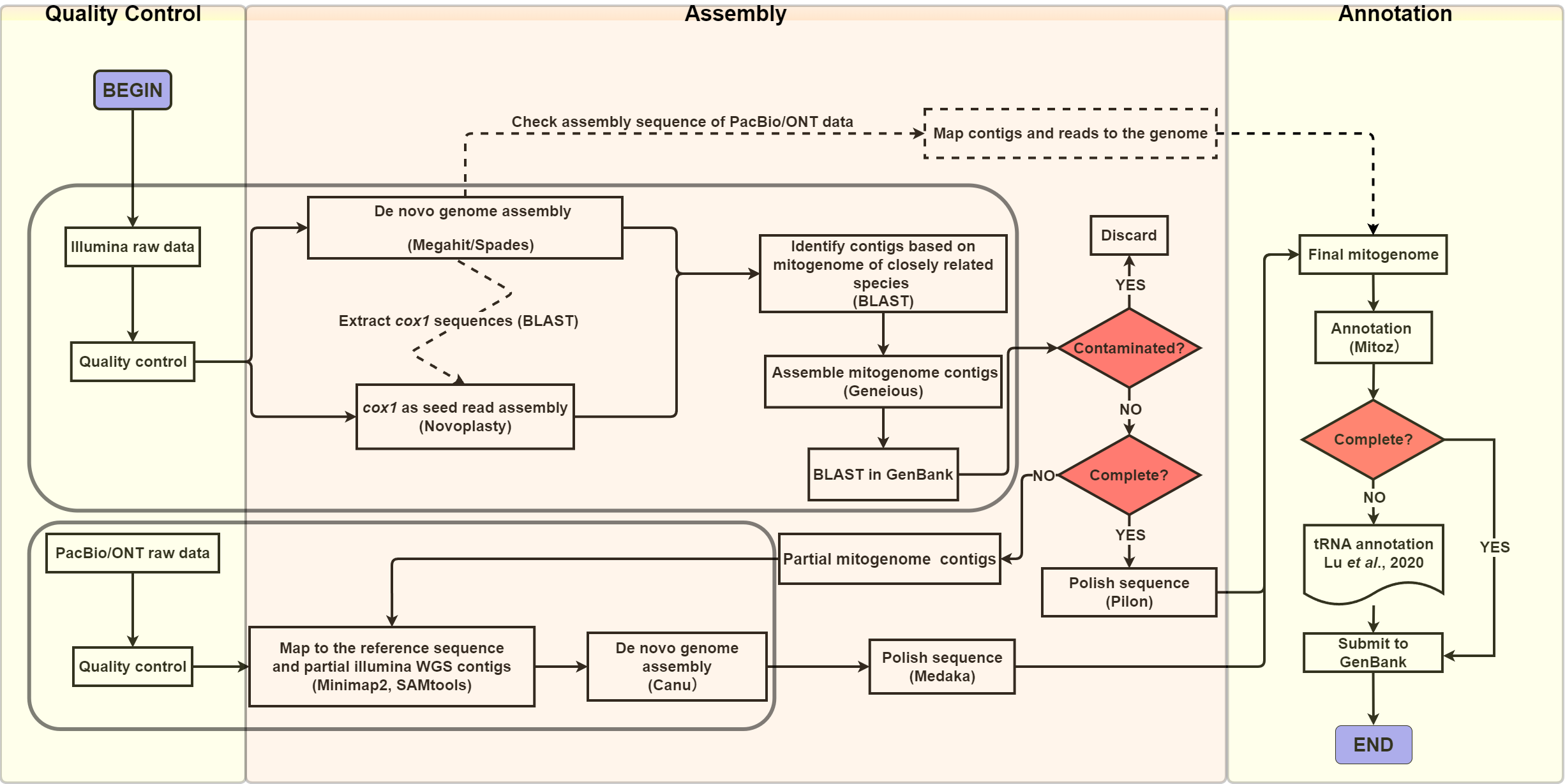

Supplement: Supplementary material 7 — Workflow chart [file zookeys-1272-047_article-178506__-s007.png]

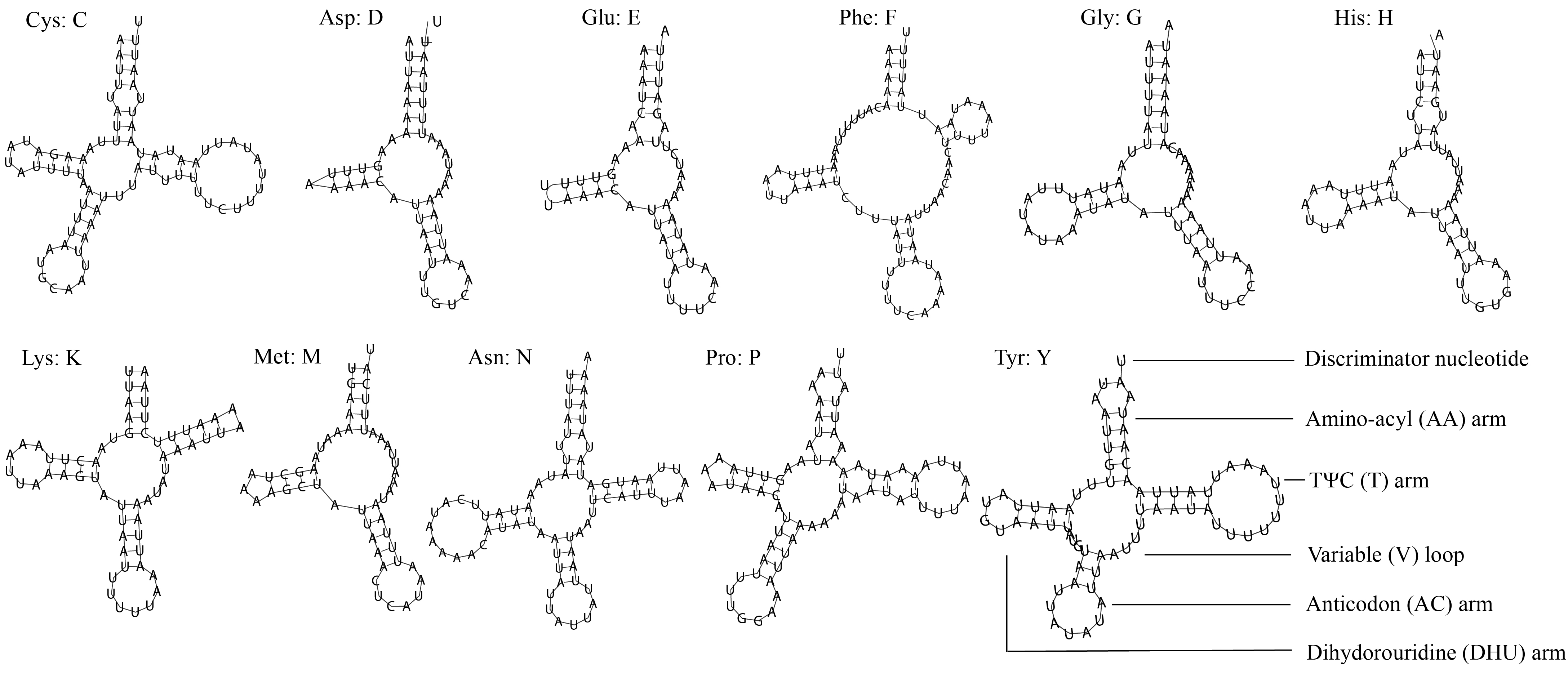

Supplement: Supplementary material 8 — Secondary structure of tRNA genes in A. yasumatsui mitochondrial genome [file zookeys-1272-047_article-178506__-s008.jpg]
